# Relative effects of location relative to the corpus luteum and lactation on the transcriptome of the bovine oviduct epithelium

**DOI:** 10.1186/s12864-019-5616-2

**Published:** 2019-03-21

**Authors:** Yann Locatelli, Niamh Forde, Helmut Blum, Alexander Graf, Benoît Piégu, Pascal Mermillod, Eckhard Wolf, Patrick Lonergan, Marie Saint-Dizier

**Affiliations:** 1 UMR85 PRC, INRA, CNRS 7247, Université de Tours, IFCE, Nouzilly, France; 2 Division of Reproduction and Early Development, Faculty of Medicine and Health Sciences, University of Leeds, Nouzilly, UK; 3 Laboratory for Functional Genome Analysis (LAFUGA), Gene Center, LMU Munich, Leeds, Germany; 4 School of Agriculture and Food Science, University College Dublin, Dublin, Ireland; 5 MNHN, Laboratoire de la Réserve Zoologique de la Haute Touche, Obterre, France; 6 Université de Tours, UFR Sciences et Techniques, Parc de Grandmont, F-37200 Tours, France

**Keywords:** Oviduct, Lactation, RNAseq, Dairy cow, Metabolism

## Abstract

**Background:**

Lactation and associated metabolic stresses during the post-partum period have been shown to impair fertility in dairy cows. The oviduct plays key roles in embryo development and the establishment of pregnancy in cattle. The aim of this study was to investigate the effects of lactation and location relative to the corpus luteum (CL) on the transcriptome of the bovine oviduct epithelium.

**Results:**

An original animal model was used. At 60 days post-partum, Holstein lactating (*n* = 4) and non-lactating (i.e. never milked after calving; *n* = 5) cows, as well as control nulliparous heifers (n = 5), were slaughtered on Day 3 following induced estrus, and epithelial samples from the oviductal ampulla and isthmus ipsilateral and contralateral to the corpus luteum (CL) were recovered for RNA sequencing. In the oviduct ipsilateral to the CL, differentially expressed genes (DEGs) were identified between heifers compared with both postpartum cow groups. However, only 15 DEGs were identified between post-partum lactating and non-lactating cows in the ipsilateral isthmus and none were identified in the ipsilateral ampulla. In contrast, 192 and 2583 DEGs were identified between ipsilateral and contralateral ampulla and isthmus, respectively. In both regions, more DEGs were identified between ipsilateral and contralateral oviducts in non-lactating cows and heifers than in lactating cows. Functional annotation of the DEGs associated with comparisons between metabolic groups highlighted a number of over-represented biological functions and cell pathways including immune response and cholesterol/steroid biosynthesis.

**Conclusions:**

Gene expression in the oviduct epithelium, particularly in the isthmus, was more affected by the location relative to the CL than by lactation at Day 3 post-estrus. Furthermore, the effect of the proximity to the CL was modulated by the metabolic status of the cow.

**Electronic supplementary material:**

The online version of this article (10.1186/s12864-019-5616-2) contains supplementary material, which is available to authorized users.

## Background

The physiological changes associated with high milk production are related to poor reproductive efficiency in commercial dairy herds [1, 2]. Decreasing (glucose, insulin-like growth factor 1) or increasing (non-esterified fatty acids, NEFA, ketone bodies) circulating metabolites during nutrient partitioning associated with low body condition score (BCS) undoubtedly play a role in determining reproductive outcome. However, the causes of infertility in dairy cattle are complex and may be attributable to compromised oocyte quality and/or a suboptimal reproductive tract environment incapable of supporting normal embryo/conceptus development or a combination of both.

Fertilization and the first 4–5 days of embryo development occur in the oviduct, which provides a unique environment in terms of metabolites, proteins, steroid hormones and ions, as compared with the uterine lumen [3–7]. The oviduct clearly plays a role in providing an appropriate environment conducive to normal embryo development and establishment of pregnancy. Data from nonsurgical flushing of single-ovulating dairy cows indicate that about 50% of embryos degenerate by Day 7 after breeding, i.e. before or just after leaving the oviduct [8, 9]. We have reported that embryo development to Day 7 following endoscopic transfer to the oviducts of postpartum lactating cows was compromised in comparison to that in the tract of nulliparous heifers [10], consistent with the previously reviewed data. More recently, age-matched postpartum primiparous dairy cows that were either milked post calving (i.e. post-partum lactating) or were dried off immediately at calving (i.e., post-partum non-lactating) were used to directly test the effects of lactation on postpartum fertility characteristics [11]. Consistent with the results of Rizos et al. [10], transfer of embryos to the oviducts of lactating cows resulted in lower blastocyst development than that observed in post-partum non-lactating cows or heifers [11], suggesting an impaired oviduct environment due to the metabolic stress associated with lactation. The endometrial transcriptome was reported to be affected by lactation on Day 17 in cyclic and pregnant Holstein cows [12] and on Day 19 in pregnant Holstein cows [13]. However, data on the effects of lactation on the oviductal environment are currently lacking.

In addition to systemic changes induced by lactation [14], normal changes in the oviductal environment in response to the formation and function of the corpus luteum (CL) may possibly be perturbed by the high energy demands associated with lactation. The concentrations of several steroid hormones in bovine oviductal tissue and oviductal fluid were reported to be higher in the ipsilateral than the contralateral side relative to ovulation [7, 15]. Moreover, transcriptomic differences between the oviduct epithelial cells ipsilateral and contralateral to the early CL have been reported [16]. However, the possible interaction between the metabolic status of the cow and the regulatory role of the proximal CL is currently not known.

In this study, we hypothesized that part of the difference in fertility between heifers or post-partum non-lactating and lactating dairy cows could be explained by differences in the transcriptome of the oviduct epithelium, associated with a compromised ability to support early embryo development. The animals used in this study were part of a large cohort previously characterized for their metabolic status [14] and included age-matched post-partum primiparous lactating or non-lactating dairy cows, and a contemporaneous group of nulliparous non-pregnant heifers. A subset of females in each group was previously used to study the effect of lactation on metabolomic profiles in the follicular fluid [14] and uterine fluid [17], as well as on gene expression in the conceptus [17] and endometrium [13] on Day 19 of pregnancy. In the current study, in an independent subset of non-inseminated animals from each of the three groups, we collected oviduct tissues on Day 3 of a synchronized estrous cycle on which we carried out RNA sequencing in order to better understand the factors contributing to reduced early embryo survival in lactating cows.

## Results

**Fig. 1:**
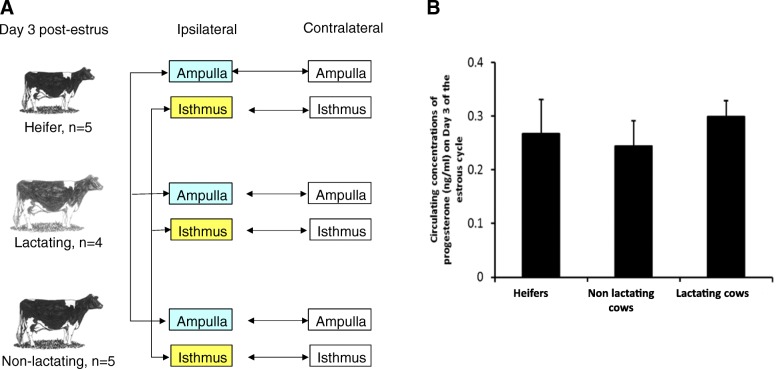
**a** Comparisons of animals and tissues performed in the RNAseq data analyses. All tissues were collected at Day 3 after estrus synchronization. Vertical arrows indicate comparisons between metabolic groups (heifer/lactating/non-lactating) in one given oviductal tissue (ampulla or isthmus) ipsilateral to the corpus luteum. Horizontal arrows indicate comparisons between ipsilateral and contralateral ampulla or isthmus in each metabolic model. **b** Serum progesterone concentrations (means ± SEM) in Holstein heifers and age-matched postpartum non-lactating (dry) and lactating cows at the time of oviduct tissue collection on Day 3 after synchronized estrus

The different groups of animals and tissues used in the RNAseq analyses are summarized in Fig. 1a.

### Plasma progesterone levels according to the metabolic status

No differences were observed in plasma concentrations of progesterone on Day 3 of the estrous cycle between heifers, post-partum non-lactating and lactating cows (Fig. 1b).

### Effect of lactation on the transcriptome of the oviduct epithelium in the ampulla and isthmus ipsilateral to the corpus luteum

The effect of the metabolic status on the oviduct epithelial transcriptome was assessed in the ampulla and isthmus ipsilateral to the CL, where fertilization and early embryo development takes place in vivo.

#### In the ampulla

**Fig. 2:**
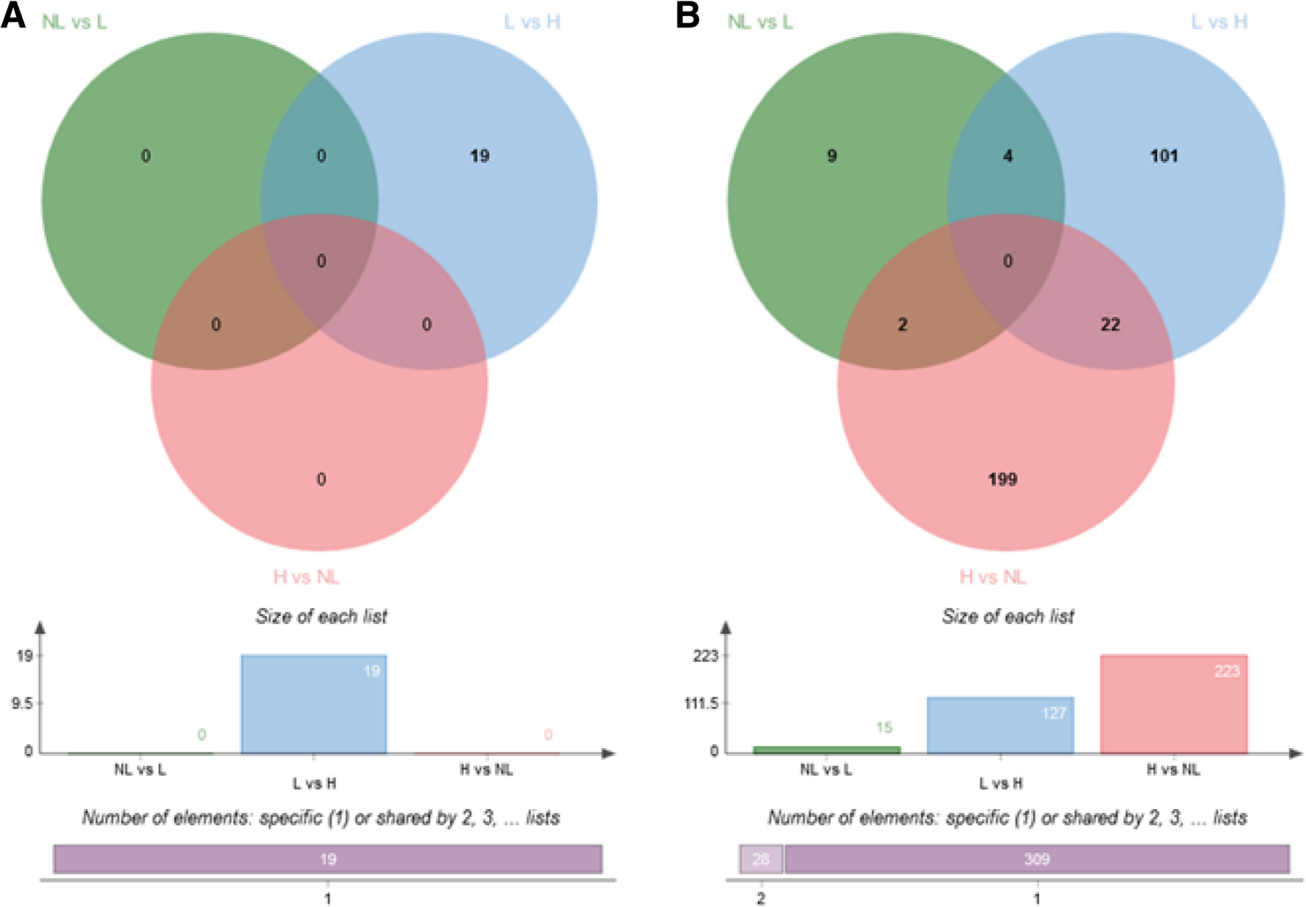
Venn diagrams of differentially expressed genes (DEGs) identified in pairwise comparisons between heifers (H) and post-partum lactating (L) and non-lactating (NL) cows in the ampulla (left diagram, **a**) and isthmus (right diagram, **b**) ipsilateral to the corpus luteum on Day 3 of the estrous cycle. The histograms below show the total number of DEGs in each group

The comparison of post-partum lactating vs. non-lactating cows and heifers vs. post-partum non-lactating cows revealed no differentially expressed genes (DEG) in the ampulla. In this region, 19 genes were differentially expressed between heifers and lactating cows (Fig. 2a; see the lists of all DEGs between metabolic groups in Additional file 1: Table S1).

#### In the isthmus

In the isthmus, only 15 DEGs were identified between post-partum lactating and non-lactating cows. However, 127 DEGs were identified between heifers and post-partum lactating cows and 223 DEGs between heifers and post-partum non-lactating cows. The overlap in DEGs between heifers vs. lactating and heifers vs. non-lactating comparisons was low as only 22 DEGs were shared between the two lists (Fig. 2b and Additional file 1: Table S1).

**Fig. 3:**
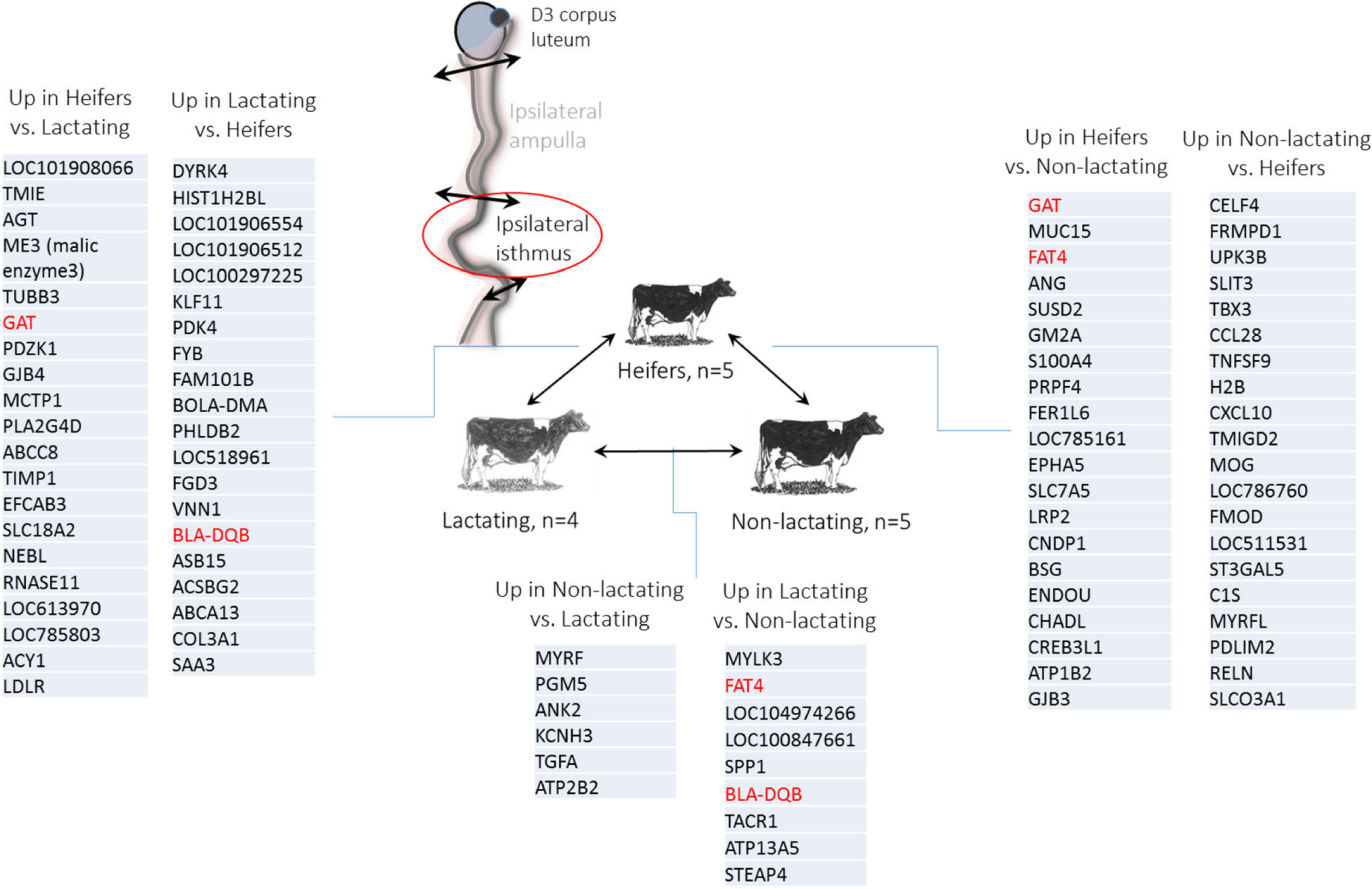
Lists of most differentially expressed genes (according to fold changes) identified in pairwise comparisons between heifers, non-lactating and lactating cows in the ipsilateral isthmus. Genes in red are shared by two comparisons (Heifers vs. Lactating and Non-lactating cows for *GAT*; Lactating vs. Heifers and Non-lactating cows for *BLA-DQB*; Non-lactating vs. Heifers and Lactating cows for *FAT4*)

Lists of the most differentially expressed genes between metabolic groups in the isthmus and their regulation (up or down) are presented in Fig. 3. Among those, *GAT* (coding for glycine N-acyltransferase) was down-regulated in both post-partum lactating and non-lactating cows compared to heifers, while *BLA-DQB* (major histocompatibility complex class II antigen) was up-regulated in lactating cows compared with both non-lactating cows and heifers. In addition, *FAT4* (FAT atypical cadherin 4) was down-regulated in non-lactating cows compared with both heifers and lactating cows (Fig. 3).

**Fig. 4:**
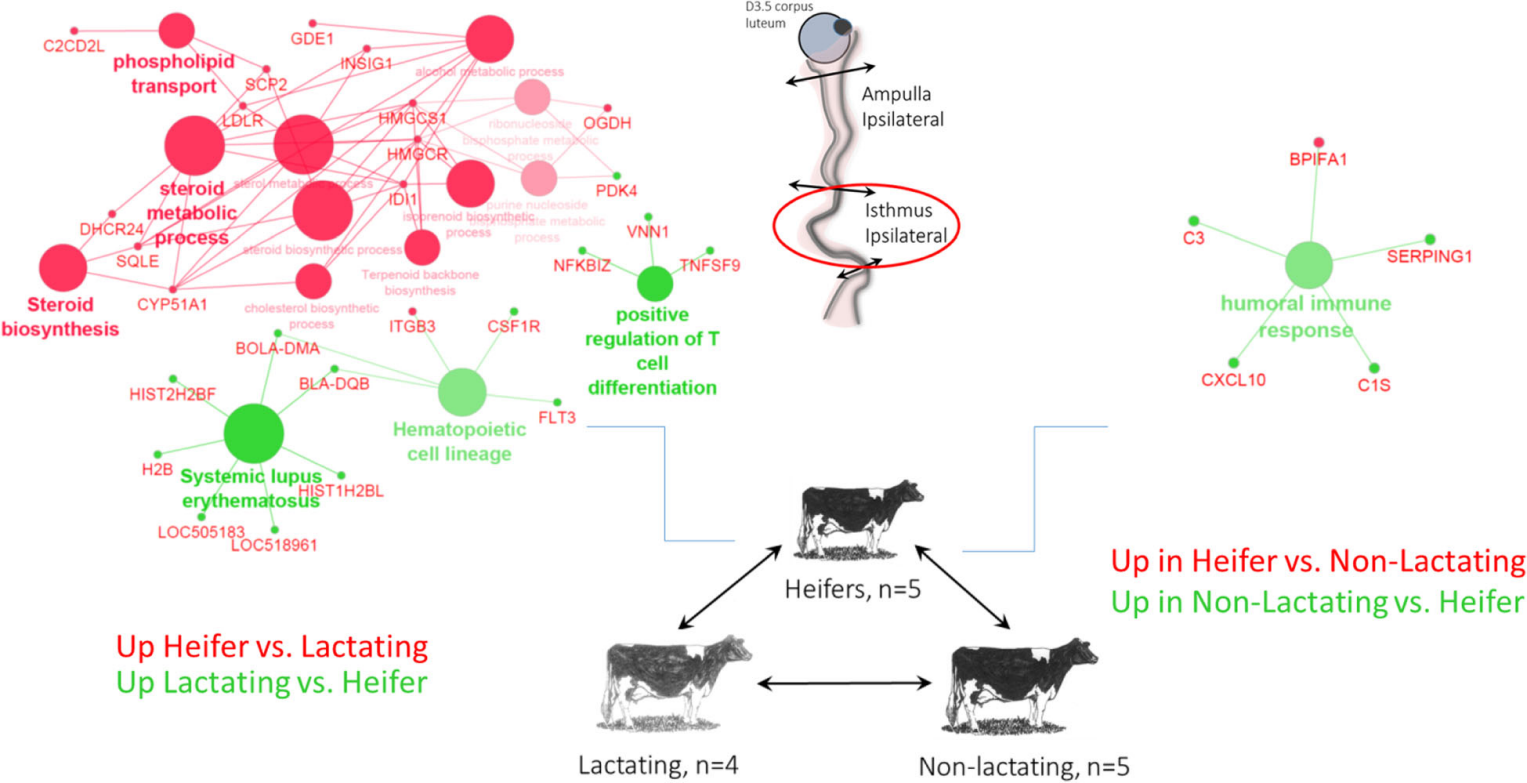
Schematic representation of Biological Process and Kyoto Encyclopedia of Genes and Genomes (KEGG) pathways/compounds associated with differentially expressed genes (DEGs) in pairwise comparison between heifers, lactating and non-lactating cows in the isthmus ipsilateral to the corpus luteum on Day 3 of the estrous cycle (Cytoscape Cluego analysis). Terms in red includes up-regulated DEGs in heifers compared with lactating (left) or non-lactating (right) cows. Terms in green includes up-regulated DEGs in lactating (left) or non-lactating (right) compared with heifers

Analysis of the gene ontology (GO) terms associated with the DEGs between heifers and lactating cows indicated several Kyoto Encyclopedia of Genes and Genomes (KEGG) pathways and Biological Process (BP) involved in steroid metabolism. GO terms such as ‘steroid biosynthesis’, ‘steroid metabolic process’ and ‘cholesterol biosynthetic process’ were associated with up-regulated genes in heifers (Fig. 4). The BP ‘pyruvate metabolic process’ was also identified with five associated genes (3 upregulated in heifers and 2 upregulated in lactating). Analysis of the GO terms associated with the DEGs between heifers and non-lactating cows indicated the ‘humoral immune response’ BP associated with up-regulated DEGs in non-lactating cows (Fig. 4; Additional file 1: Table S1).

### Effect of location relative to the corpus luteum on the transcriptome of oviduct epithelium in the ampulla and isthmus

**Fig. 5:**
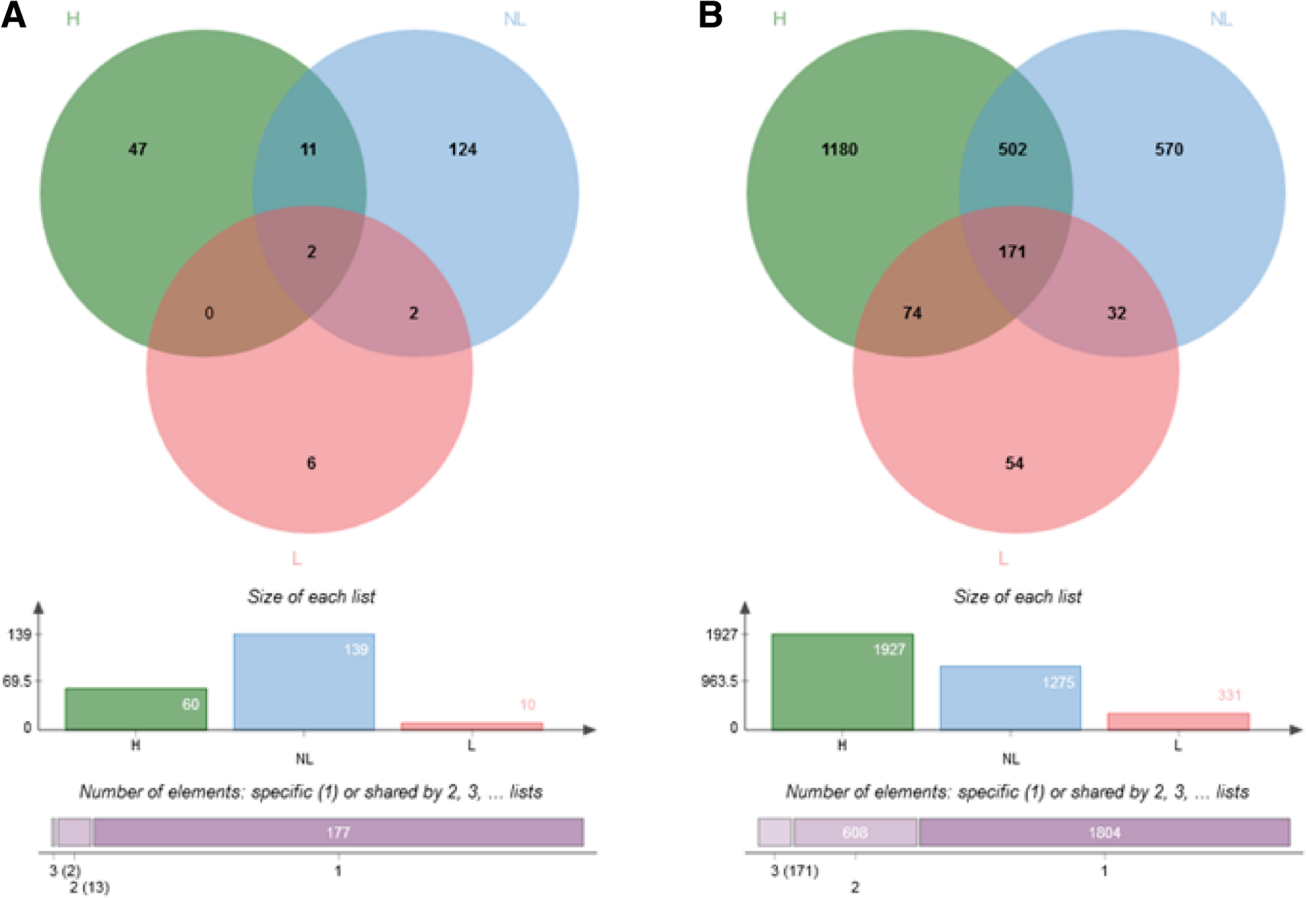
Venn diagrams of differentially expressed genes (DEGs) identified between ipsilateral and contralateral ampullas (left diagram, **a**) and isthmus (right diagram, **b**) in heifers (H), post-partum lactating (L) and non-lactating (NL) cows on Day 3 of the estrous cycle. The histograms below show the total number of DEGs in each group

Overall, gene expression in both the ampulla and isthmus was more affected by the location relative to the CL than by lactation. Irrespective of the metabolic status, the number of DEGs between ipsilateral and contralateral cells was higher in the isthmus than in the ampulla (2583 vs. 192 DEGs, Fig. 5). Furthermore, in both regions, the lowest number of DEGs was identified in lactating cows compared with heifers and non-lactating cows (i.e. the highest number of DEG was identified by comparing heifers with non-lactating cows).

#### In the ampulla

**Fig. 6:**
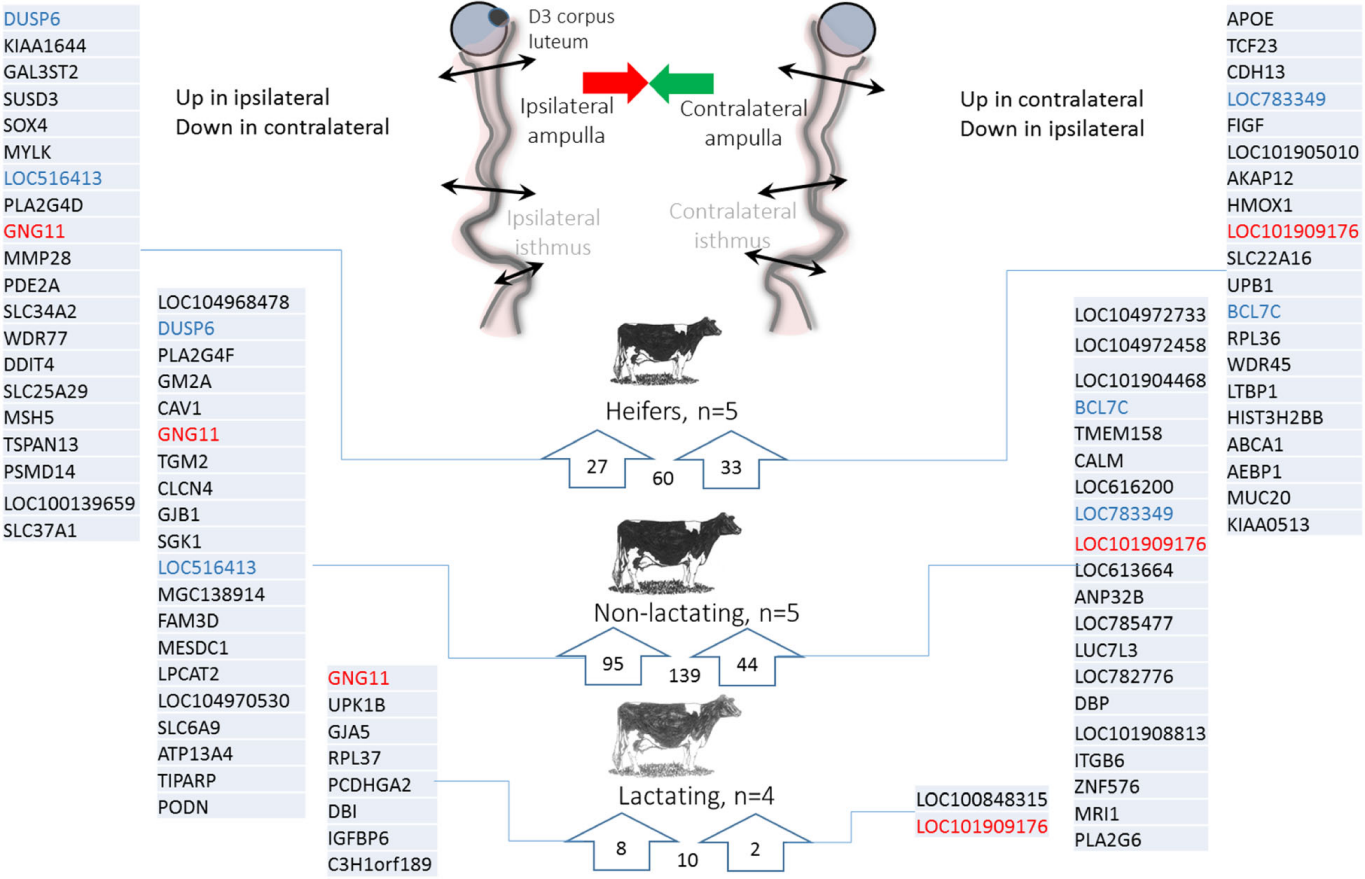
Total number and top-20 (according to fold change) differentially expressed genes (DEGs) identified between ipsilateral and contralateral ampullas in heifers, non-lactating and lactating cows on Day 3 of the estrous cycle. DEGs upregulated in the ampulla ipsilateral to the corpus luteum (CL) (or downregulated in the contralateral side) are listed on the left. DEGs upregulated in the ampulla contralateral to the CL (or downregulated in the ipsilateral side) are listed on the right. Genes in red and blur are shared by the three metabolic groups, and heifers and non-lactating cows, respectively

Overall, 192 DEGs were identified between the ipsilateral and contralateral ampullas, most of which were in the non-lactating group (139 DEGs of which 124 were specific to this group; see Additional file 2: Table S2 for all DEGs between the ipsilateral and contralateral ampullas). Furthermore, a low proportion (7.8%) of those DEGs was shared between metabolic groups (11 genes shared between heifers and non-lactating; two between non-lactating and lactating; none between lactating and heifers and two shared by the three groups; Fig. 5a). Of the 192 DEGs identified, 73 were up- and 119 were down-regulated in the ipsilateral ampulla compared to the contralateral side, with relatively low fold changes (91% of the 192 DEGs with a fold change < 2). The top 20 up- and down- DEGs in each metabolic group are presented in Fig. 6.

Given the low number of DEGs in the lactating group, GO analyses were performed only in the two other groups. Detailed lists of BP associated with up- and down-regulated genes in the ampulla are provided in Additional file 2: Table S2. In the non-lactating group, four KEGG pathways such as ‘Arginine and proline metabolism’ or ‘Valine, leucine and isoleucine degradation’ involving up-regulated genes in the ipsilateral ampulla were identified. Among BP identified, ‘steroid metabolic process’ and ‘cholesterol homeostasis’ were associated to up -regulated genes in ipsilateral ampulla. In the ampulla from heifers, only the ‘Cholesterol metabolism’ KEGG pathway was identified, associated with *ABCA1* and *APOE* which were down-regulated in the ipsilateral side.

#### In the isthmus

**Fig. 7:**
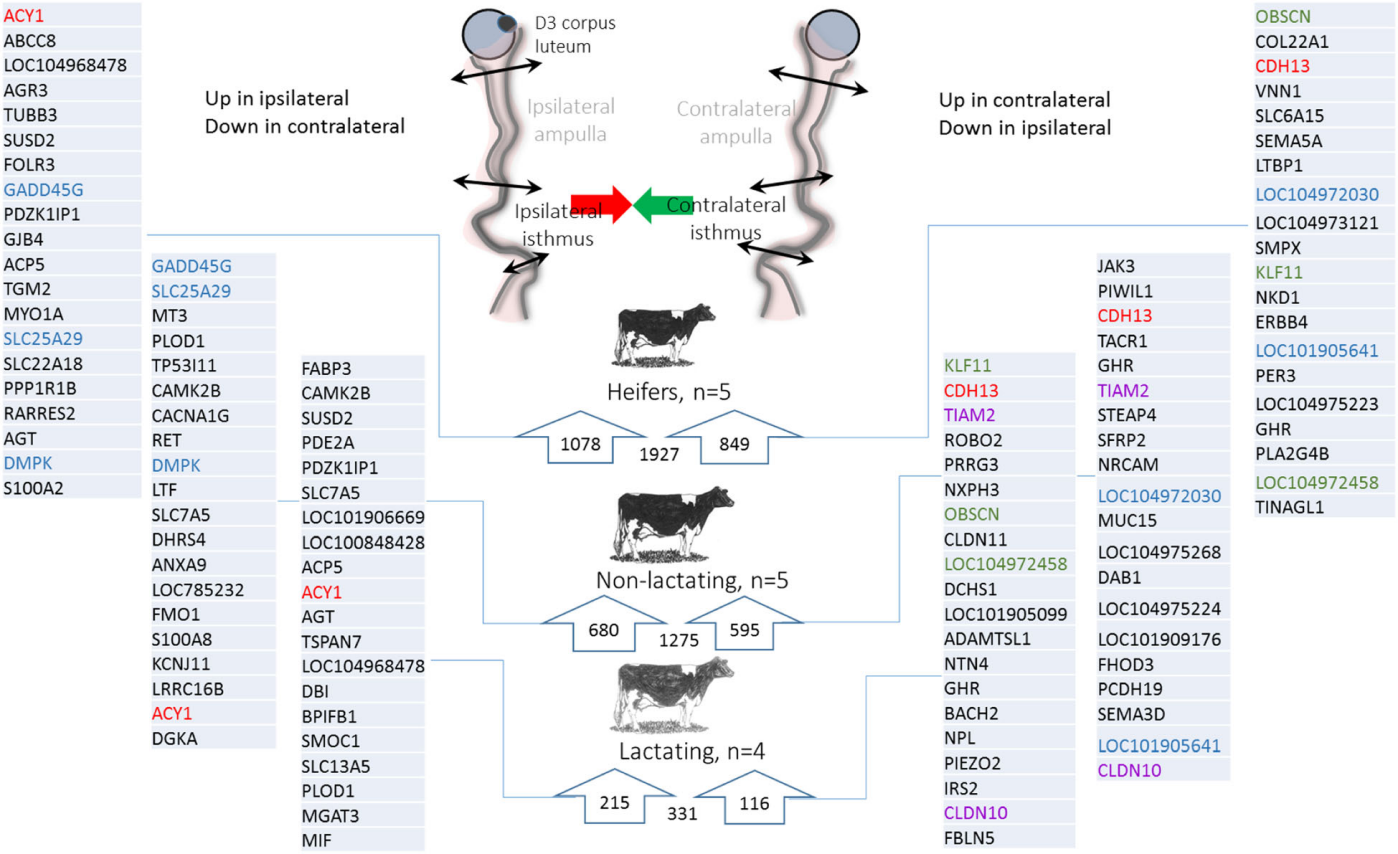
Total number and top-20 (according to fold changes) differentially expressed genes (DEGs) identified between ipsilateral and contralateral isthmus in heifers, non-lactating and lactating cows on Day 3 of the estrous cycle. DEGs upregulated in the isthmus ipsilateral to the corpus luteum CL (or downregulated in the contralateral side) are listed on the left. DEGs upregulated in the isthmus contralateral to the CL (or downregulated in the ipsilateral side) are listed on the right. Genes in red, blue, green and purple are shared by the three metabolic groups, heifers and non-lactating cows, heifers and lactating cows, and non-lactating and lactating cows, respectively

A total of 2583 DEGs were identified when comparing the ipsilateral and contralateral isthmus, with a low to moderate overlap between groups of animals (502 DEGs shared between heifers and non-lactating cows; 74 between heifers and lactating cows; 32 between non-lactating and lactating cows; 171 shared by the three groups; see Fig. 5b and Additional file 3: Table S3). The heifers group was the most affected by the proximity of the CL (1927 DEGs), followed by the post-partum non-lactating (1275) and lactating (331) groups. Overall, 1162 genes were up-regulated and 1421 down-regulated in the ipsilateral isthmus, with a higher degree of differential expression than in the ampulla (max fold changes of 3.9 for up-regulated genes and 6.1 for down-regulated genes; see top-20 up and down DEGs for the isthmus in Fig. 7 and Additional file 3: Table S3).

**Fig. 8:**
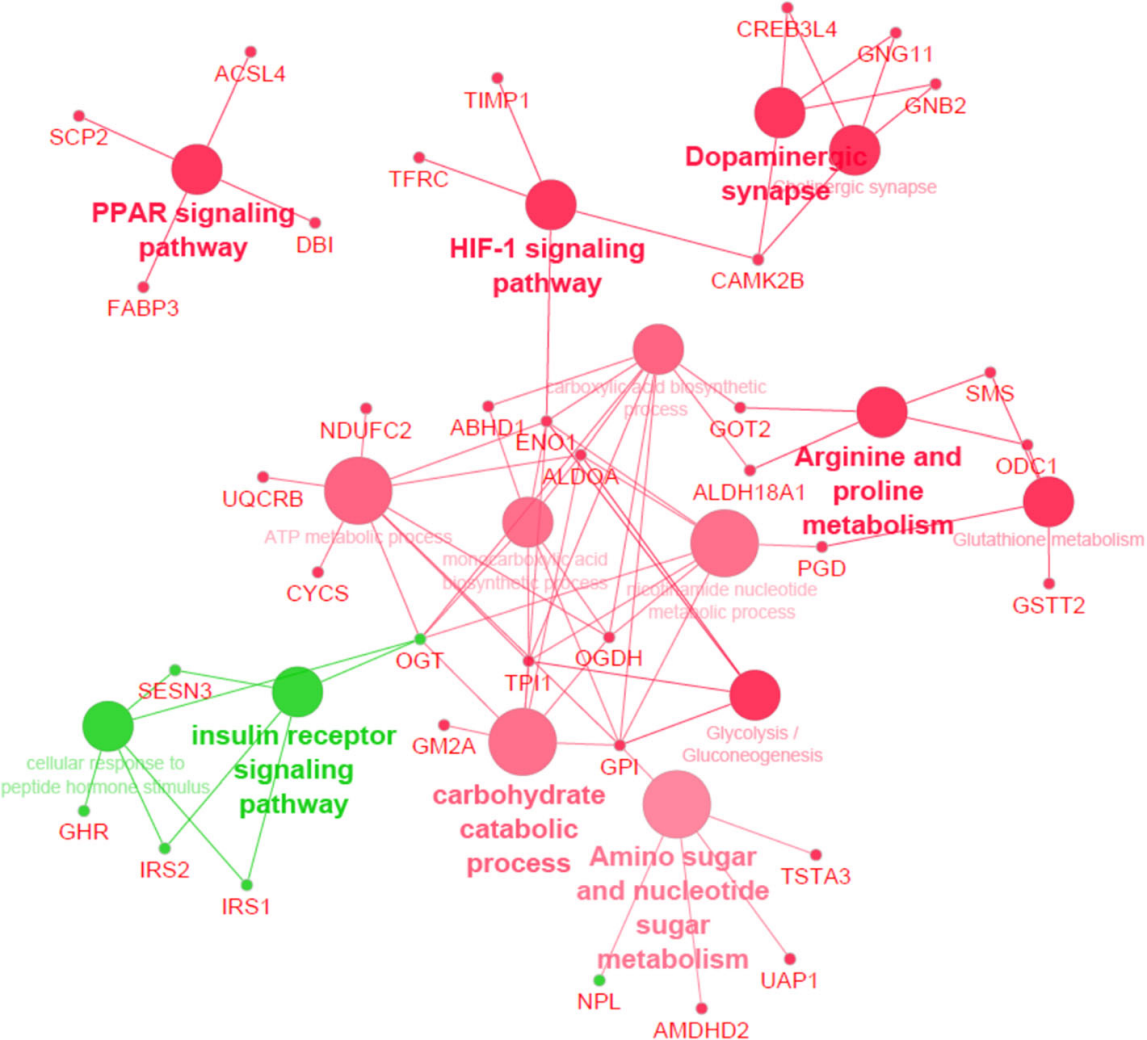
Schematic representation of Biological Process and Kyoto Encyclopedia of Genes and Genomes (KEGG) pathways/compounds associated with DEGs shared by heifers, post-partum non-lactating and lactating cows when comparing ipsilateral and contralateral isthmus at Day 3 of the estrous cycle (Cytoscape ClueGO analysis). Networks including genes up-regulated in ipsilateral isthmus are in red while networks including genes up-regulated in contralateral isthmus (or down-regulated in ipsilateral isthmus) are represented in green

A high number of BP and KEGG pathways were identified for each metabolic group. Most of these GO terms included genes specifically up-regulated in the ipsilateral isthmus (Additional file 3: Table S3). Lastly, when considering the 171 DEGs shared between the three metabolic groups (of which 115 were up-regulated in the ipsilateral and 56 up-regulated in the contralateral isthmus), six KEGG pathways were identified, including ‘Amino sugar and nucleotide sugar metabolism’, ‘PPAR signalling pathway’, ‘HIF-1 signaling pathway’, ‘Arginine and proline metabolism’, ‘Glutathione metabolism’ and ‘Glycolysis/gluconeogenesis’ (Fig. 8 and Additional file 3: Table S3).

## Discussion

By comparing post-partum non-lactating and lactating Holstein cows, this study addressed the effect of lactation on gene expression in the oviduct. The main findings were that on Day 3 of the estrous cycle when, in pregnant animals the embryo would be at the 8-cell stage and undergoing embryonic genome activation, 1) gene expression in the oviduct was more affected by the location relative to the CL than by lactation, irrespective of the oviduct region; 2) the isthmus, where the embryo would be located at this stage in pregnant animals, was more affected by the metabolic status of the cow and the location relative to the CL than the ampulla; 3) a higher number of DEGs according to the proximity of the CL were identified in heifers and non-lactating cows than in lactating cows, suggesting an interaction between lactation and the regulatory role of the proximal CL on gene expression in oviduct epithelium.

### Effect of metabolism on oviduct epithelial gene expression

Results from recent studies in which bovine embryos were transferred into the oviduct of animals of different metabolic status indicate that the oviductal environment is compromised by lactation [10, 11]. In the present study, the direct comparison between oviduct epithelial cells from post-partum lactating and non-lactating cows on Day 3 of the estrous cycle identified only 15 DEGs in the isthmus and none in the ampulla. This may be related to the variability in gene expression profiles observed within groups. Nonetheless, the results suggest that lactation does not dramatically affect the transcriptome of the oviduct at this stage. Similarly, RNA-sequencing performed on the endometrium from lactating and non-lactating cows on Day 19 of pregnancy failed to demonstrate a major effect of lactation on uterine gene expression; the number of DEGs identified between lactating and non-lactating cows was much lower than that identified between heifers and both groups of cows [13]. However, we cannot exclude a modification by lactation of the composition of the oviductal fluid, in which the early embryo develops. Several amino acids were reported to be differentially abundant in the uterine luminal fluid ipsilateral to the CL recovered from heifers compared with lactating and non-lactating cows on Day 19 of pregnancy [17]. Oviductal fluid is a complex and dynamic fluid resulting from the ultrafiltration of the circulating blood, de novo synthesis from oviduct epithelial cells and potential input from the preovulatory follicle. The negative energy balance associated with lactation in the post-partum period is well established in dairy cattle [2]. Up to 60 days post-partum, at which time the oviducts were collected in the current study, important modifications in plasma and intra-follicular concentrations of numerous metabolites and hormones, that could contribute to the alteration of the oviductal environment, were reported between non-lactating and lactating cows [11, 14, 18].

Investigation of the effect of metabolism on the oviduct transcriptome allowed to the identification of DEGs and associated signalling pathways in the ipsilateral oviduct. Overall, the enrichment of GO functions observed in comparisons between metabolic groups may underline some interesting differences in tissue physiology. Genes such as *FAT4 (*FAT atypical cadherin 4), *SPP1* (osteopontin), *PGM5* (phosphoglucomutase 5), *STEAP4* (STEAP4 metalloreductase), *MYLK3* (myosin light chain kinase 3) and *TGFA* (transforming growth factor alpha) that were regulated in the isthmus of lactating cows compared to non-lactating counterparts could represent interesting candidates. The up-regulation of *SPP1* in the isthmus of lactating cows was, however, unexpected. Indeed, the addition of *SPP1*-derived protein, osteopontin, to culture medium was shown to improve in vitro fertilization and embryo development in the porcine and bovine species [19, 20].

When comparing heifers with lactating cows, the expression of genes involved in the metabolism of pyruvate and glucose, two metabolites essential for embryo development, was also affected in the ipsilateral isthmus. *PDK4* and *PRKAG3* were found up-regulated in lactating cows whereas *HK1*, *OGDH* and *ME3* were found up-regulated in heifers. This may reflect some possible consequences of the negative energy balance associated with lactation on oviduct metabolism. *PDK4* (pyruvate dehydrogenase kinase 4) plays a key role in the pyruvate dehydrogenase complex by switching from glucose to fatty acid degradation. *HK1* (hexokinase 1) phosphorylates glucose to produce glucose-6-phosphate, the first step in most glucose metabolism pathways. *OGDH* (oxoglutarate dehydrogenase) encodes a subunit that catalyzes the oxidative decarboxylation of alpha-ketoglutarate to succinyl-CoA. *ME3* (NADP-dependent malic enzyme, mitochondrial) plays a pivotal role in allowing malate oxidative decarboxylation to pyruvate. Taken together, these data suggest different orientation in oviduct tissue metabolism according to the metabolic status of the animal itself. Differences in pyruvate metabolism pathway in oviductal cells may in turn affect concentrations of metabolites in the oviductal fluid. This is of particular interest for the early embryo which switches from an oxidative metabolism (using pyruvate and lactate as the main sources of energy) to a glycolytic metabolism during its travel through the oviduct [21].

Numerous genes involved in cholesterol metabolism and steroid biosynthesis were also identified as differentially expressed between heifers and lactating cows in the ipsilateral isthmus, as well as in the ipsilateral ampulla. Recently, cholesterol biosynthesis was identified as a key pathway altered with modification of estrogen/progesterone environment in the bovine oviduct [22]. Taken together, these data suggest that heifers and lactating cows may either differ in their steroid secretion at the ovarian level or in steroid diffusion to oviduct. While ovarian steroids are well known to regulate numerous functions in the oviduct, few studies examined the ability of oviductal cells to synthesize steroid hormones. In equines, oviductal explants were able to produce high amounts of progesterone in vitro and the expression of several steroidogenic enzymes were evidenced in oviductal cells, suggesting oviduct steroidogenic capacities [23, 24]. Further research is needed to examine if the bovine oviduct is able to metabolize cholesterol/steroids and whether this ability differs between heifers and lactating cows.

Gene expression analysis in the ipsilateral isthmus from heifers compared to non-lactating cows allowed the identification of a different set of DEGs and associated BP. Interestingly, BP such as ‘humoral immune response’ (including *BPIFA1*, *C1S*, *C3*, *CXCL10* and *SERPING1* genes) and ‘complement activation’ (*C1S*, *C3* and *SERPING1*) were found up-regulated in the isthmus of non-lactating cows, suggesting potential differences in the modulation of the maternal immune system between the two groups. In the mouse and human, complement C3 has been shown to be expressed in the oviduct epithelium [25, 26]. During early pregnancy, the oviduct and the early embryo collaborate to convert C3 into its derivative iC3b, which has an embryotrophic effect [26, 27]. The expression of *C1S* and *SERPING1* has been documented in the endometrium of Day 18 pregnant cows and the simultaneous upregulation of *SERPING1*, encoding a protein known as C1 inhibitor, has been proposed as a mechanism to modulate the maternal immune system and protect the embryo from the complement [28]. Whether these differences in gene expression could be involved in differences in embryo survival between heifers and cows previously observed by others [10, 11] remains to be determined.

### Effect of the location relative to the CL on oviduct gene expression

Differences in gene expression were identified between epithelial cells derived from ipsilateral and contralateral oviducts in heifers, lactating and non-lactating cows. These differences may reflect effects of the developing CL on the proximal oviduct. Ipsilateral and contraletaral oviducts were reported to differ in their number of mucosal folds [29], hormonal content [7, 15], protein content [6] and gene expression [16, 29] in the post-ovulatory period in cows. Similar to the latter studies, our experimental model cannot differentiate the potential effects of the presence of a non-fertilized oocyte and its surrounding cumulus cells on the ipsilateral oviduct. Indeed, the presence of gametes (spermatozoa) or developing embryos after multiple embryo transfer has been reported to modify the oviductal transcriptome in vivo [30, 31]. However, one of these studies [31] was in pigs, a litter-bearing species, while in the other, the presence of a single embryo and spermatozoa after artificial insemination did not have any effect on the oviduct transcriptome on Day 3 after estrus in heifers [30]. In our study, samples were collected 3 days after estrus, i.e. around 2 days after ovulation; based results from [30], it is unlikely that a single unfertilized oocyte would have detectable effects on the oviductal transcriptome.

A total number of 2775 DEGs were detected between ipsilateral and contralateral oviducts, irrespective of the oviduct segment and metabolic group. This effect of the side of ovulation on oviductal gene expression is in agreement with a previous transcriptomic study comparing ipsilateral and contralateral epithelial cells at Day 3.5 post-estrus in heifers [16]. Although in limited numbers, the majority of DEGs identified in the latter by suppressive subtractive hybridization (27/35) were up-regulated in the ipsilateral oviduct [16], while in the present study, numbers of up- and down-regulated DEGs in both regions of the oviduct were similar (73 up- and 119 down-regulated in the ampulla and 1162 up- and 1421 down-regulated in the isthmus).

Irrespective of the metabolic group, many more DEGs were identified according to the side of ovulation in the isthmus than in the ampulla (2583 and 192, respectively). This is in contrast to a previous study by Maillo et al. (2016), in which the proximity of the CL did not have any effect on the transcriptome of the isthmus at Day 3 post-estrus in both cyclic and pregnant heifers. In that study, microarray hybridization was used and the oviducts were collected from crossbred beef heifers [32]. The differences in the technique and breed may explain such differences in the results.

Local countercurrent transfer of ovarian steroids from the ovary to the ipsilateral oviduct has been reported in many mammals including cattle [33]. In particular, in the immediate post-ovulation period in cattle, concentrations of progesterone in the oviductal fluid [7] as well as of prostaglandins E_2_ and F_2α_ in oviductal tissues [15] were reported to be significantly higher in the ipsilateral than in the contralateral side. Several recent studies in cattle have reported differences in oviductal gene expression related to variations in the sex steroid hormone environment or after treatment of oviduct epithelial cells with estradiol or progesterone [22, 29, 34–37]. Thus, differences in gene expression between ipsilateral and contralateral oviducts may reflect the asymmetry in endocrine signals produced by the ipsilateral ovary. However, the reason why the isthmus was more affected than the ampulla by the proximity of the ovary is not clear. Data on ovarian steroid concentrations in ampulla and isthmus taken separately are currently not available. Both regions of the oviduct receive blood from tubal and uterine branches of the arterial ovary [38] and it is likely that the dynamic flow of the oviductal fluid prevents contrasted differences between the two regions. Interestingly, the gene coding for the progesterone receptor (*PGR*) was down-regulated in the ipsilateral compared to the contralateral isthmus in both heifers and lactating cows whereas *PGR* was not regulated in the ampulla. It could be speculated that at least part of the transcriptomic differences related to the side of ovulation between ampulla and isthmus at Day 3 were due to differences in progesterone responsiveness.

The regulatory role of the proximity of the CL on the oviduct transcriptome varied according to the metabolic status of the cow. Indeed, irrespective of the oviduct region, the comparison between ipsilateral and contralateral oviducts revealed many more DEGs in both heifers and non-lactating cows than in lactating cows (1987 and 1414 vs. 341 DEGs, respectively), suggesting that lactation impaired or inhibited local regulatory signals emitted by the ipsilateral ovary. Previous studies using intra-oviductal transfer of embryos showed that less embryos developed to the blastocyst stage in lactating cows compared with heifers [10] and non-lactating cows [11], showing that lactation impaired the ability of the oviduct to support embryo development. Taken together, these results suggest that there may be a link between the ability of the oviduct, particularly the isthmus, to respond to local ovarian signals and the ability to sustain embryo development. How these metabolic changes may alter the local regulatory mechanisms of the ipsilateral oviduct remain however to be explored.

## Conclusions

The detrimental effect of lactation on early embryo survival reported in previous studies [10, 11] could not be explained by a major alteration in the oviduct gene expression at Day 3 of the estrous cycle. However, location relative to the CL had a strong impact on oviduct gene expression, in particular in the isthmus, at this stage. The regulatory role of the CL was stronger in heifers and non-lactating cows than in lactating cows, suggesting an impairment in the ability of the ipsilateral CL to regulate oviduct gene expression in lactating cows, likely associated with the altered metabolic status.

## Methods

This study was part of a larger project examining the influence of metabolic status on a variety of reproductive parameters.

### Animals

The animals used in this study were part of a larger cohort. In total, 40 pregnant primiparous Holstein cows and 11 nulliparous Holstein heifers of similar estimated breeding value (EBV) were enrolled. Immediately after calving, cows were either dried off (i.e., never milked, *n* = 20) or milked twice daily (n = 20), as previously described [11, 14]. Depending of the question addressed, some animals were used for collection of follicular tissue and oocytes [14] and some for collection of uterine tissue and conceptuses after embryo transfer [13, 17]. For the present study, a subset of non-inseminated (i.e., cyclic) females from each group were slaughtered for oviduct collection at Day 3 after synchronized estrus. At approximately 60 days post-partum (dpp), the estrous cycles of non-lactating (*n* = 5) and lactating cows (*n* = 4), and nulliparous heifers (n = 5) were synchronized by insertion of a controlled intra-vaginal drug releasing device (CIDR, Pfizer Animal Health, Sandwich, Kent, UK) containing 1.38 g of P4 for 8 days (Fig. 1). One day prior to CIDR removal, all animals received a 2-ml intramuscular (i.m.) injection of a prostaglandin F2 alpha analogue (PG: Estrumate, Intervet, Dublin, Ireland; equivalent to 0.5 mg Cloprostenol) to regress the endogenous CL. Thirty six hours after CIDR removal, each animal received a 2.5-ml i.m. injection of Receptal (Intervet, Dublin, Ireland: equivalent to 0.012 mg buserelin). On Day 3 following estrus, all animals (heifers and cows) were slaughtered at a commercial abattoir.

#### Sample processing

Reproductive tracts were stored on ice prior to sample collection and were processed on-site within 15 min of slaughter. Stage of cycle was confirmed by the presence of a fresh CL (corpus haemorrhagicum) on one ovary. Oviducts ipsilateral and contralateral to the side of CL were dissected between isthmus and ampulla regions. In each oviduct segment, epithelial cells were collected by gentle scraping, immediately snap frozen and stored at − 80 °C until processing.

#### Measurement of serum progesterone concentrations

Jugular blood samples were collected before slaughter and stored at 4 °C for 24 h, spun at 1500 g at 4 °C for 20 min and the serum supernatant decanted and stored at − 20 °C prior to analysis. Serum samples were analysed for P4 concentrations as previously described [39] using the Coat-A-Count solid phase radioimmunoassay Progesterone kit (Siemens Medical Solutions Diagnostics, Los Angeles, CA, USA) with an assay sensitivity of 0.03 ng/ml. The inter-assay coefficients of variation (% CV) were 10.1, 11.9 and 1.7% for the low, medium and high quality control samples and the low, medium and high intra-assay CVs were 13.4, 6.1 and 5.4%, respectively.

### Transcriptomic analysis

#### RNA-sequencing and data analysis

Oviduct epithelial cells recovered from the ampulla and isthmus ipsilateral and contralateral to the CL were lysed with Trizol™ according to the manufacturer’s instructions. After precipitation, RNA quality and quantity was confirmed using the Agilent Bioanalyser system and all RNA samples used for RNA sequencing had an RNA Integrity Number (RIN) of 7.9 or greater. Starting from total RNA, stranded RNA sequencing libraries were constructed with the Encore Complete RNA-Seq™ library system of NuGEN. This protocol requires a minimum of 100 ng of total RNA and enriches for non-rRNA during cDNA synthesis. All libraries were sequenced on an Illumina HiSeq 1500 generating between 28 and 67 million 100 bp single-end reads per library.

First the Illumina adapter sequences were removed from the 3′-end using a custom trimming script. Another tailored custom tool was used to trim poly-A tails from the 3′- and 5′-end of each read with a seed length of 6 nt. Reads with a length of below 25 nt after clipping were filtered out. Reads were mapped with Tophat2 (v.2.0.13) [40] to the bovine reference genome Btau_4.6.1 (BosTau7) with default settings based on the gene annotation (UCSC: bosTau7) from Illumina’s iGenomes Project. Transcript abundance was counted per gene using htseq-count (v.0.6.1) [41] with strand specificity (−s yes), intersection-strict mode (−m intersection-strict) and with the GFF attribute set to ‘gene’ (−i gene). The underlying gene annotation was the same as used for the mapping.

Differences in transcript abundance were identified with the Bioconductor package DESeq2 (v.1.6.3) [42]. The fitType was set to ‘parametric’. A false discovery rate (FDR) of 0.05 was used as threshold for significance of differential transcript abundance for the comparisons heifer vs. non-lactating and heifer vs. lactating cows.

Data were analyzed in two steps. Transcriptomic differences in the oviduct epithelium (isthmus and ampulla) were compared (i) in the oviduct ipsilateral to the CL (where the embryo develops if fertilization occurs) between the three groups (heifers, postpartum lactating, postpartum nonlactating) and (ii) between ipsilateral and contralateral oviducts. Venn diagrams were generated for genes with *P* < 0.001 from all three comparisons using the web tool JVenn [43].

### Gene ontology analysis

Gene ontology (GO) analysis was performed separately on DEG lists identified in the ampulla and isthmus according to location relative to the CL (within group, including DEGs shared between groups) and between groups with a *P* < 0.05 using Cytoscape version 3.7.0 and ClueGO/CluePedia version 2.5.3 plugins in the open source Cytoscape (cystoscape.org) platform [44]. The ClueGO plugin allows to visualize the non-redundant biological terms in a functionally grouped network. The CluePedia plugin works in conjunction with ClueGO to associate genes and proteins potentially associated to networks based on in silico and/or experimental information [45]. Background lists for this analysis includes only genes detected in the ampulla or isthmus with a median of reads count > 1. This median was calculated with all samples in the same organ to generate two distinct custom reference sets (isthmus and ampulla). The DEG lists generated were entered as up- and down-regulated in separate clusters. Ontology/Pathway identification was based on GO Biological Process (BP) and/or Kyoto Encyclopedia of Genes and Genomes pathways (KEGG pathways) using GO terms fusion except if specified otherwise. The retained criteria included a GO tree level > 3, a Kappa score > 0.4 and a minimum number of 3 genes for all analyses except for the comparison between ipsilateral and contralateral ampullae from heifers, for which a minimum of 2 genes was applied (due to the total low number of DEG identified in this comparison). Only GO terms/pathways with P < 0.05 were considered.

## Additional files

Additional file 1: **Table S1.** Exhaustive and top-20 lists of up- and down-regulated genes in post-partum lactating (L) vs. non-lactating (NL) cows, heifers (H) vs. L, and H vs. NL comparisons in the ampulla and isthmus ipsilateral to the corpus luteum on Day 3 of the estrous cycle. Lists of associated Gene Ontology (GO) terms are provided for the H vs. NL and H vs. NL comparisons in the isthmus. (XLSX 106 kb)

Additional file 2: **Table S2.** Exhaustive lists of up- and down-regulated genes in ipsilateral vs. contralateral ampulla in post-partum lactating (L), non-lactating (NL) cows and heifers (H) on Day 3 of the estrous cycle. Lists of associated Gene Ontology (GO) terms are provided for ipsilateral vs. contralateral ampullas in NL and H. (XLSX 231 kb)

Additional file 3: **Table S3.** Exhaustive and top-20 lists of up- and down-regulated genes in ipsilateral vs. contralateral isthmus in post-partum lactating (L), non-lactating (NL) cows and heifers (H) on Day 3 of the estrous cycle. Lists of associated Gene Ontology (GO) terms are provided for all comparisons. The Biological Process (BP) and Kyoto Encyclopedia of Genes and Genomes (KEGG) pathways/compounds shared between the three groups are also provided. (XLSX 603 kb)

## Abbreviations

BP: biological process
CL: corpus luteum
DEGs: differentially expressed genes
GO: gene ontology
KEGG: Kyoto encyclopedia of genes and genomes
RIN: RNA integrity number
